# Breastfeeding patterns among parturients with diabetes: A secondary analysis of the MOMPOD randomized clinical trial

**DOI:** 10.1002/pmf2.12040

**Published:** 2025-01-28

**Authors:** Minhazur Sarker, Marni B. Jacobs, Kim Boggess, Ashley N. Battarbee, Jerrie Refuerzo, Noelia Zork, Kacey Eichelberger, Celeste Durnwald, Mark Landon, Kjersti Aagaard, Kedra Wallace, Christina Scifres, Sherri Longo, Alison Stuebe, Gladys A. Ramos, the MOMPOD Study Consortium

**Affiliations:** ^1^ University of California San Diego San Diego California USA; ^2^ University of North Carolina at Chapel Hill School of Medicine Chapel Hill North Carolina USA; ^3^ University of Alabama at Birmingham Heersink School of Medicine Birmingham Alabama USA; ^4^ University of Texas Health Houston McGovern Medical School Houston Texas USA; ^5^ Columbia University Irving Medical Center New York New York USA; ^6^ University of South Carolina School of Medicine Greenville/Prisma Health‐Upstate Greenville South Carolina USA; ^7^ University of Pennsylvania Perelman School of Medicine Philadelphia Pennsylvania USA; ^8^ The Ohio State University College of Medicine and Wexner Medical Center Columbus Ohio USA; ^9^ HCA Houston Healthcare – Texas Maternal Fetal Medicine Houston Texas USA; ^10^ University of Mississippi Medical Center Jackson Mississippi USA; ^11^ Indiana University School of Medicine Indianapolis Indiana USA; ^12^ Ochsner Health New Orleans Louisiana USA

**Keywords:** breastfeeding, early diabetes, lactogenesis, metformin, pregestational diabetes

## Abstract

**Introduction:**

Insulin resistance is associated with decreased milk supply in lactating people. Metformin is hypothesized to increase breast milk production by decreasing insulin resistance, suggesting use may increase breastfeeding success. We aimed to determine the association between metformin use during pregnancy and breastfeeding initiation and continuation.

**Methods:**

This was a secondary analysis of the MOMPOD randomized controlled trial of metformin versus placebo in addition to insulin therapy among pregnant people with type 2 diabetes and early diabetes. We included parturients who delivered a living neonate, received at least one dose of study drug or placebo, endorsed an intention to breastfeed, and completed a breastfeeding survey. Breastfeeding intentions and breastfeeding outcomes were collected utilizing a breastfeeding questionnaire at 24–30 weeks and 30 days postpartum, respectively. The primary outcome was breastfeeding at 30 days postpartum defined by exclusive or partial breastfeeding. Secondary outcomes included immediate breastfeeding defined as any breastfeeding during the postpartum hospital admission until at least postpartum day 3, onset of lactogenesis (days), breast and bra size, and breastfeeding challenges. Baseline characteristics and outcomes were compared using chi‐square, *t*‐test, or Wilcoxon tests, as appropriate.

**Results:**

Among the 794 women randomized and receiving either placebo or metformin in the primary trial, 378 (47.6%) met inclusion criteria with 194 (51.3%) in metformin and 184 (48.7%) in placebo groups. There were no significant differences in baseline characteristics. Immediate breastfeeding was comparable between groups (91.1% vs. 88.9%, *p* = 0.53) and there was no difference in onset of lactogenesis. Breastfeeding rates were lower among all parturients 30 days postpartum and there was no difference between metformin and placebo groups (76.0% vs. 66.7%, *p* = 0.11). Also, there were no differences in partial or exclusive breastfeeding, breast cup or bra size, or breastfeeding challenges.

**Conclusion:**

Our data suggest no association between metformin use and breastfeeding patterns in those with type 2 or early diabetes in pregnancy. Antepartum metformin should not be recommended solely to improve breastfeeding success.

## 1 INTRODUCTION

Breastfeeding has been associated with decreased maternal risk of developing diabetes [1, 2, 3], hypertension [1, 2, 4, 5], hyperlipidemia [1, 2], and cardiovascular disease [6, 7]. Along with maternal benefits of breastfeeding, there are also numerous benefits for the newborn including prevention of childhood obesity [8, 9, 10]. Given these benefits, the World Health Organization, the American College of Obstetricians and Gynecologists, and the American Academy of Pediatrics all recommend exclusive breastfeeding for the first six months of life and continued breastfeeding for at least the first two years of life [11, 12, 13, 14].

In birthing parents with diabetes, who are at a higher risk for short‐ and long‐term adverse health outcomes, the benefits of breastfeeding also include reduction of neonatal hypoglycemia and onset of childhood diabetes [15, 16, 17, 18]. Despite these benefits, breastfeeding rates in parturients with diabetes are less than their counterparts without diabetes [19, 20, 21, 22, 23]. Challenges to both initiation and continuation of breastfeeding among parturients with diabetes include cesarean delivery, operative vaginal delivery, obesity, presence of hypertensive disorders, delayed lactation or impaired lactogenesis, and neonatal factors such as macrosomia, congenital anomalies, prematurity, respiratory distress syndrome, neonatal hypoglycemia, and neonatal ICU admission all of which are more common among patients with diabetes [19, 24, 25–32, 33, 34, 35]. Furthermore, pregnancy complications can independently affect overall maternal confidence and breastfeeding efficacy [24, 36–39]. Given these significant barriers, finding effective ways to support breastfeeding success in pregnancies complicated by diabetes is critically important.

Insulin during pregnancy and lactation stimulates the mammary gland to increase milk production in both animal and human models [40, 41, 42, 43]. Thus, insulin resistance has been identified as a contributing factor interfering with successful breastfeeding initiation and continuation [44]. Given these relationships, metformin has been hypothesized as a treatment to improve lactogenesis and breastfeeding rates in pregnancies complicated by diabetes. Multiple small pilot studies have been performed to determine metformin's clinical utility for breastfeeding both antepartum and postpartum but robust studies are lacking [45, 46, 47]. We aimed to determine the association between metformin use for the treatment of diabetes in pregnancy and breastfeeding patterns among parturients at 30 days postpartum.

## 2 MATERIALS AND METHODS

This is a secondary analysis of the MOMPOD study (Medical Optimization of Management of Overt Type 2 Diabetes in Pregnancy), a multicenter, double‐blinded, randomized controlled trial of metformin versus placebo added to insulin for the treatment of pre‐existing type 2 diabetes or diabetes diagnosed in early pregnancy [48]. Participants aged 18–45 with either type 2 diabetes or diabetes diagnosed in early pregnancy carrying a singleton gestation were randomized 1:1 between 10w0d and 22w6d to the addition of either metformin or placebo twice daily across 17 clinical sites in the United States from June 2017 and November 2021 (enrollment was suspended from April 2020 to September 2020 due to the COVID‐19 pandemic). Randomization was stratified by clinical site, gestational age at randomization, and type of diabetes (type 2 diabetes or diabetes in early pregnancy). The Institutional Review Board at each site approved the primary study and separate approval was not required for the secondary analysis. The primary randomized controlled trial was registered on ClinicalTrials.gov with Identifier NCT02932475.

Type 2 diabetes was confirmed by medical chart review and diabetes in early pregnancy was diagnosed by any of the following prior to 23‐week gestation: (1) one‐step testing (75‐g oral glucose tolerance test with ≥ 1 abnormal value), (2) two‐step testing (positive 50‐g oral glucose challenge test followed by 100‐g oral glucose tolerance test with ≥ 2 abnormal values by Carpenter–Coustan criteria [49]), (3) glycated hemoglobin ≥ 6.5%, (4) fasting capillary blood glucose ≥ 126 mg/dL, or (5) random capillary blood glucose ≥ 200 mg/dL [50, 51, 52, 53]. All enrolled participants were treated with weight‐based insulin including long‐acting and/or rapid‐acting formulations. Participants were instructed to take 500 mg metformin or identical placebo capsules twice daily for 1 week then increase to 1000 mg twice daily if well tolerated. Participants were asked to perform self‐monitoring of blood glucose with a glucometer by measuring fasting glucose in the morning aiming for a goal of 70–95 mg/dL and postprandially with a goal of < 140 mg/dL for 1 h postprandial and < 120 mg/dL for 2 h postprandial. Postpartum management of diabetes was at the discretion of the primary provider.

During the late second to third trimester between 24 and 30 weeks’ gestation, participants were given a breastfeeding survey to determine their breastfeeding intentions (Figure S1). During the delivery hospitalization, immediate breastfeeding initiation defined as any breastfeeding during the postpartum hospital admission until at least postpartum day 3 was assessed. A follow‐up survey was administered via a phone call at 30 days postpartum to determine newborn breastfeeding patterns (exclusive vs. partial breastfeeding), if any formula supplementation was used, and bra size and bra cup before and after pregnancy. Among participants who stopped breastfeeding, further questions were asked regarding perception of factors that contributed to breastfeeding success, timing of breastfeeding cessation and formula initiation, and whether they subjectively felt they met their intended breastfeeding goals. Postpartum visits were conducted per primary site provider preferences.

In this secondary analysis described herein, we included parturients who delivered a live neonate, received at least one dose of the study treatment drug (metformin or placebo), endorsed an intention to breastfeed, and completed the breastfeeding surveys. Intention to breastfeed was further characterized by calculating each participant's breastfeeding intention score using the validated Infant Feeding Intentions scale [54]. The Infant Feeding Intentions scale is a well‐validated 16‐point scale to determine intent to breastfeed with a higher score being more consistent with exclusive breastfeeding. The primary outcome was breastfeeding at 30 days postpartum defined by ongoing exclusive or partial breastfeeding. Secondary outcomes included immediate breastfeeding, onset of lactogenesis (days), breast and bra sizes, and breastfeeding challenges among those who stopped breastfeeding. With respect to breastfeeding challenges, we included neonatal latching, maternal supply, breastmilk alone not satisfying the newborn, neonatal weight gain concern, and maternal pain. As there is no standardized method to calculate breast cup size, we defined breast cup size similarly to one prior study that converted standard United States breast cup sizes to quantitative values based on the following method: A = 1, B = 2, C = 3, D = 4, DD/E = 5, DDD/F = 6 [47].

In the primary MOMPOD study, the data safety and monitoring board recommended cessation of the study due to metformin futility. A total of 831 participants were randomized and 794 participants took at least 1 dose of the study medication, either metformin or placebo [48]. Despite study cessation, 378 participants met final inclusion criteria for this secondary analysis. With 180 participants per group in this secondary analysis, we determined a priori that we would have 80% power to detect approximately a 30%–60% increase in breastfeeding with metformin if the baseline rate of breastfeeding is estimated to be 20%–40% in the placebo group by 30 days postpartum. Our baseline placebo group breastfeeding rate was derived from findings in the breastfeeding literature for pregnancies complicated by diabetes [23, 46, 55]

All statistical analyses were performed on SAS 9.4. Baseline characteristics and outcomes were compared between treatment groups using chi‐square, student's T‐test, or Wilcoxon tests, as statistically appropriate. We planned to perform an adjusted analysis only for differences in baseline characteristics but our cohorts were similar. A *p*‐value of 0.05 was considered significant. There was no imputation for missing data and no adjustment for multiple comparisons.

## 3 RESULTS

**FIGURE 1:**
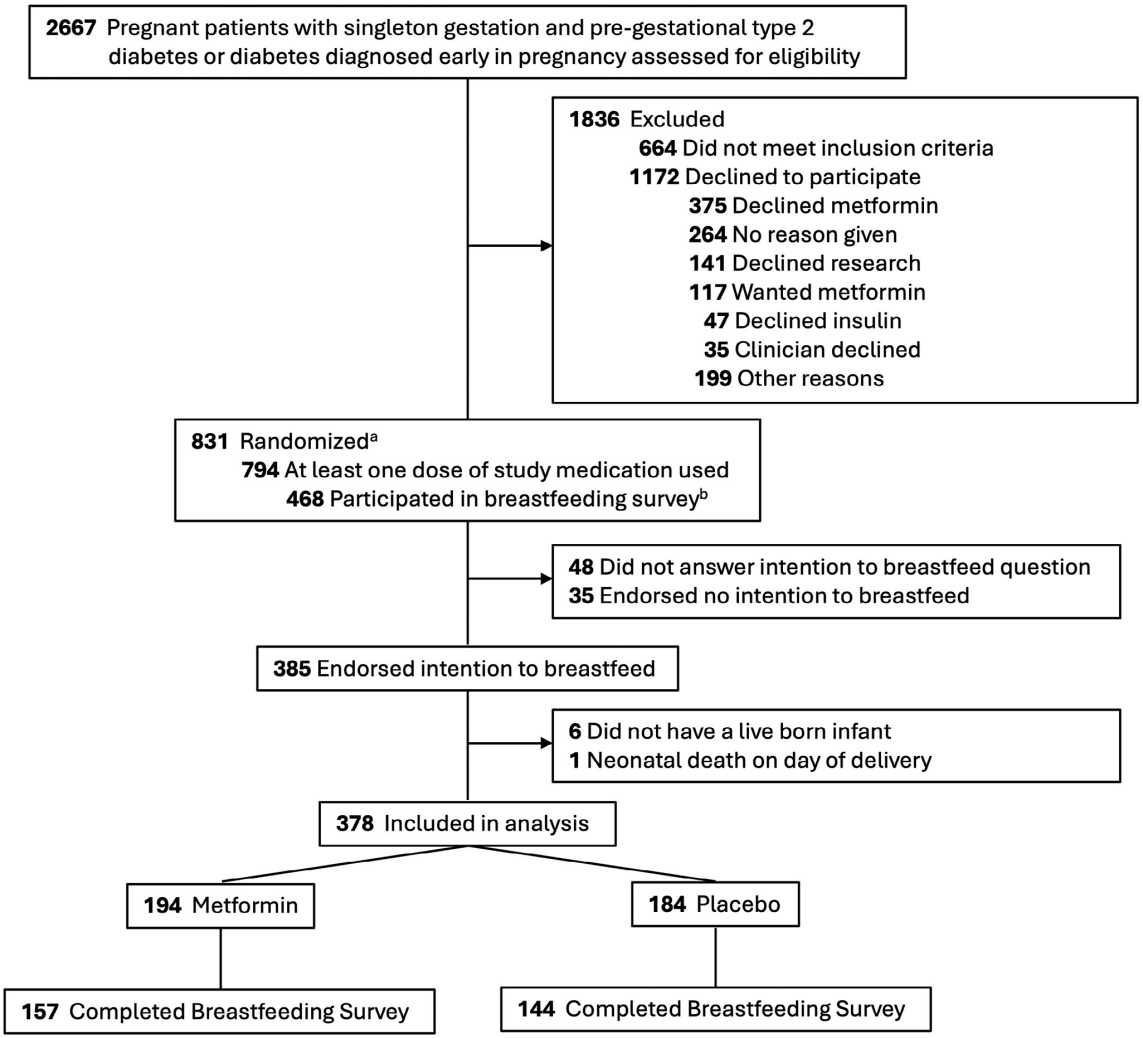
Study flow chart. ^a^Randomization was stratified by clinical site, gestational age at randomization (< 18 weeks vs. ≥18 weeks), and timing of diabetes diagnosis (pregestational or during pregnancy). ^b^Participants who discontinued the study drug were included in the primary analysis.

Of the 794 participants that received at least one dose of the metformin or placebo in addition to their insulin regimen by the time of trial cessation, 468 (59%) completed the breastfeeding intention questionnaire, 385 (48.5%) endorsed an intention to breastfeed, and 378 (47.6%) met all inclusion criteria for our planned secondary analysis with 194 (51.3%) in the metformin group and 184 (48.7%) in the placebo group (Figure 1). In the metformin group, immediate postpartum responses were collected from 157 (80.9%) of participants and 121 (62.4%) of participants completed the 30‐day postpartum survey. In the placebo group, immediate postpartum responses were collected from 144 (78.3%) of participants and 120 (65.2%) of participants completed the 30‐day postpartum survey. With respect to race and ethnicity, our cohort represents a diverse population (Table 1). There was no difference in the baseline breastfeeding intention score between the two groups (Table 1). There were no statistically significant baseline differences noted between groups with respect to maternal age, maternal body mass index, nulliparity, parity, type of diabetes, hypertensive disorder, gestational age at randomization, gestational age at delivery, mode of delivery, neonatal birthweight, or NICU admission rates (Table 1).

**TABLE 1:** Baseline characteristics of the study groups.

		Comparison groups		
	Overall *n* = 378 (100.0%)	Metformin *n* = 194 (51.3%)	Placebo *n* = 184 (49.7%)	*p*‐value
Breastfeeding intention score				0.78
Mean (SD)	9.0 (2.5)	9.0 (2.5)	8.9 (2.5)	
Median (Q1, Q3)	9.5 (7, 11)	9.5 (7, 11)	9.0 (7, 11)	
Maternal age, mean (SD)	33.2 (5.6)	32.9 (5.2)	33.6 (5.9)	0.28
Hispanic, *n* (%)	195 (51.6)	99 (51.0)	96 (52.2)	0.82
Race, *n* (%)				0.58
White	174 (46.0)	86 (44.3)	88 (47.8)	
Black/African American	103 (27.2)	55 (28.4)	48 (26.1)	
Asian	7 (1.9)	4 (2.1)	3 (1.6)	
Native Hawaiian/Pacific Islander	2 (0.5)	1 (0.5)	1 (0.5)	
American Indian/Alaska Native	3 (0.8)	2 (1.0)	1 (0.5)	
Mixed	4 (1.1)	4 (2.1)	0 (0.0)	
Unknown/refused	85 (22.5)	42 (21.7)	43 (23.4)	
Maternal BMI at enrollment, mean (SD)	36.7 (8.5)	36.4 (7.5)	37.0 (9.4)	0.53
BMI category at enrollment, *n* (%)				0.80
BMI < 30	80 (21.2)	39 (20.3)	41 (22.7)	
BMI 30–40	179 (47.4)	95 (49.5)	84 (46.4)	
BMI ≥ 40	114 (30.2)	58 (30.2)	56 (30.9)	
Gestational age at randomization, mean (SD)	16.8 (3.5)	16.8 (3.4)	16.9 (3.7)	0.77
Type of Insurance				0.05
Medicaid/State	278 (73.5)	136 (73.9)	142 (73.6)	
Private	75 (19.8)	41 (22.3)	34 (17.6)	
Military	1 (0.3)	1 (0.5)	0 (0.0)	
None	23 (6.1)	6 (3.3)	17 (8.8)	
Nulliparity, *n* (%)	39 (10.3)	14 (8.7)	25 (15.7)	0.06
Parity, median (Q1, Q3)	2 (1, 3)	2 (1, 3)	2 (1, 3)	0.75
Pre‐existing diabetes, *n* (%)	278 (73.5)	143 (73.7)	135 (73.4)	0.94
Hypertensive disorders at time of delivery, *n* (%)				0.78
None	184 (48.7)	94 (48.7)	90 (49.7)	
Chronic hypertension	38 (10.1)	22 (11.4)	16 (8.8)	
Gestational hypertension	39 (10.3)	18 (9.3)	21 (11.6)	
Preeclampsia	113 (29.9)	59 (30.6)	54 (29.8)	
Gestational age at delivery, mean (SD)	37.1 (2.2)	37.2 (2.3)	37.0 (2.1)	0.27
Mode of delivery, *n* (%)				0.70
Vaginal delivery (including spontaneous, vacuum, and forceps assisted deliveries)	134 (35.4)	67 (34.7)	67 (36.6)	
Cesarean delivery	242 (64.0)	126 (65.3)	116 (63.4)	
Birthweight, mean (SD)	3184.0 (726.0)	3131.0 (695.7)	3240.1 (754.4)	0.15
Admission to NICU	143 (37.8)	65 (33.5)	78 (42.4)	0.08
NICU length of stay, median (Q1, Q3)	8 (3, 23)	9 (3, 29)	7 (3, 15)	0.51

In our primary outcome analysis, rates of breastfeeding at 30 days postpartum did not reach statistical significance (metformin 76.0% vs. placebo 66.7%, difference of 9.3%, 95% CI −2.0% to 20.7%, *p* = 0.11) and there were no notable differences between exclusive and partial breastfeeding (Table 2). Additionally, the rates of immediate breastfeeding did not differ between the metformin and placebo groups (91.1% vs. 88.9%, *p* = 0.53) (Table 2). Similarly, there were no differences noted in onset of lactogenesis between exposure groups (Table 2). Of note, 87.9% in the metformin group and 93.1% in the placebo group utilized formula supplementation at some point during the postpartum period (Table 2). Finally, we noted no differences in breast cup or bra sizes before pregnancy or postpartum (Table 2).

**TABLE 2:** Breastfeeding outcomes associated with metformin.

		Comparison groups		
	Overall *n* = 378 (100.0%)	Metformin *n* = 194 (51.3%)	Placebo *n* = 184 (49.7%)	*p*‐value
Any immediate breastfeeding in the hospital^a^	271/301 (90.0)	143/157 (91.1)	128/144 (88.9)	0.53
Actively breastfeeding at 30 days postpartum^b^	172/241 (71.4)	92/121 (76.0)	80/120 (66.7)	0.11
Currently breastfeeding^b^				0.84
No	98/270 (36.3)	48/140 (34.3)	50/130 (38.5)	
Yes, exclusive	41 (15.2)	22/140 (15.7)	19/130 (14.6)	
Yes, partial	131/270 (48.5)	70/140 (50.0)	61/130 (47.7)	
Onset of lactogenesis (days)				0.55
1 day or less	65/270 (24.1)	36/140 (25.7)	29/130 (22.0)	
2 days	54/270 (20.0)	26/140 (18.6)	28/130 (21.2)	
3 days	57/270 (21.1)	30/140 (21.4)	27/130 (20.5)	
4 days	36/270 (13.3)	18/140 (12.9)	18/130 (13.6)	
More than 4 days	51/270 (18.9)	28/140 (20.0)	23/130 (17.4)	
Milk never came in	9/270 (3.3)	2/140 (1.4)	7/130 (5.3)	
Ever fed formula, *n* (%)	245/271 (90.4)	123/140 (87.9)	122/131 (93.1)	0.14
Stopped breastfeeding or transitioned to primarily formula feeding by the postpartum visit, *n* (%)	101/378 (26.7)	47/194 (24.2)	54/184 (29.3)	0.26
Breast cup size, mean (SD)^c^				
Breast cup size before pregnancy	3.6 (1.4)	3.6 (1.5)	3.5 (1.3)	0.39
Breast cup size postpartum	3.8 (1.6)	3.8 (1.7)	3.7 (1.4)	0.68
Bra size (cm), mean (SD)^d^				
Bra size before pregnancy	38.6 (3.7)	38.5 (3.5)	38.8 (3.9)	0.55
Bra size postpartum	38.9 (3.3)	39.0 (3.6)	38.7 (3.0)	0.43

*Note*: Given the variable completion of each survey element, denominators for each measure are noted in the table for clarity.

^a^ Initial postpartum responses were collected from 157/194 (80.9%) in metformin group and by 144/184 (78.3%) in the placebo group.

^b^ Postpartum survey questions pertaining to the primary outcome were completed by 121/157 (77.1%) in metformin group and by 120/144 (83.3%) in the placebo group.

^c^ Standard breast cup size was converted to quantitative variable (A = 1, B = 2, C = 3, D = 4, DD/E = 5, DDD/F = 6) and data were obtained from participants with any breastfeeding.

^d^ Data were obtained from participants with any breastfeeding.

There were similar proportions of participants who reported issues and stopped breastfeeding or used primarily formula feeding in the metformin and placebo groups (47 (24.2%) and 54 (29.3%), *p* = 0.26, respectively) (Table 3). Among these participants, there were no differences in the weeks at formula initiation or breastfeeding cessation (Table 3). With regard to participants’ perception of factors leading to breastfeeding success, maternal milk supply was felt to be “very important” by 57% and 59% of participants in the metformin and placebo groups, respectively, which was not significantly different. Similarly, when asked about the perceived importance of neonatal latching on breastfeeding success, 48.9% in the metformin group and 40.7% in the placebo group felt it was a “very important” contributor. Conversely, the majority of participants answered that newborn weight gain and maternal breast pain were “not at all important” or “not very important” to breastfeeding success (Table 3). Finally, among participants who stopped breastfeeding, a minority of them felt that they achieved their breastfeeding goals at 30 days postpartum (18.5% in metformin vs. 11.4% in the placebo group (*p* = 0.43)).

**TABLE 3:** Issues with breastfeeding among those who stopped breastfeeding or transitioned to primarily formula feeding.

		Comparison groups		
	Overall *n* = 101 (100.0%)	Metformin *n* = 47 (24.2%)	Placebo *n* = 54 (29.3%)	*p*‐value
Contribution of factors to breastfeeding success				
Maternal supply				0.97
Not at all important	24 (23.8)	11 (23.4)	13 (24.5)	
Not very important	5 (5.0)	2 (4.3)	3 (5.7)	
Somewhat important	13 (12.9)	7 (14.9)	6 (11.3)	
Very important	58 (57.4)	27 (57.4)	31 (58.5)	
Neonatal latching				0.49
Not at all important	35 (34.7)	14 (29.8)	21 (38.9)	
Not very important	7 (6.9)	2 (4.3)	5 (9.3)	
Somewhat important	14 (13.9)	8 (17.0)	6 (11.1)	
Very important	45 (44.6)	23 (48.9)	22 (40.7)	
Baby not gaining enough weight				0.52
Not at all important	45 (44.6)	24 (51.1)	21 (39.6)	
Not very important	12 (11.9)	5 (10.6)	7 (13.2)	
Somewhat important	13 (12.9)	4 (8.5)	9 (17.0)	
Very important	30 (29.7)	14 (29.8)	16 (30.2)	
Painful				0.84
Not at all important	56 (55.4)	26 (55.3)	30 (56.6)	
Not very important	22 (21.8)	10 (21.3)	12 (22.6)	
Somewhat important	6 (5.9)	4 (8.5)	2 (3.8)	
Very important	16 (15.8)	7 (14.9)	9 (17.0)	
Breastmilk alone did not satisfy baby				0.84
Not at all important	28 (27.7)	16 (34.0)	12 (28.3)	
Not very important	7 (6.9)	3 (6.4)	4 (7.6)	
Somewhat important	16 (15.8)	6 (12.8)	10 (18.9)	
Very important	46 (45.5)	22 (46.8)	24 (45.3)	
Weeks at breastfeeding cessation, mean (SD)	2.8 (2.0)	3.0 (2.4)	2.5 (1.5)	0.32
Weeks at formula initiation, mean (SD)	1.4 (0.9)	1.3 (0.8)	1.4 (0.9)	0.80
Met breastfeeding goal (yes), *n* (%)	9/62 (14.5)	5/27 (18.5)	4/35 (11.4)	0.43

## 4 DISCUSSION

Findings from our study suggest no association between antepartum metformin use and breastfeeding outcomes in participants with type 2 diabetes or diabetes diagnosed in early pregnancy, although our study has limited power to detect smaller differences. We observed no differences in breastfeeding initiation or continuation to 30 days postpartum. Additionally, we found no differences between treatment groups in time to milk production or breast cup or bra size suggesting no association between metformin and lactogenesis. Among survey responders, our rate of immediate breastfeeding was high in both groups with a cumulative rate of 90.0%, which is similar to the immediate breastfeeding rates among other populations with diabetes intending to breastfeed [23, 55, 56]. Among survey responders, our active breastfeeding rate at the postpartum visit of 76.0% and 66.7% in the metformin and placebo groups, respectively, was comparatively high relative to the literature. This increased baseline breastfeeding success may be due to a combination of increased lactational support at our large academic medical centers and the Hawthorne effect, whereby participant actions are biased given their participation in a research study.

The use of metformin to enhance breastfeeding has been studied previously in smaller pilot studies. One study was a secondary analysis of a randomized controlled multicenter study on metformin versus placebo from first trimester until delivery in 186 participants with polycystic ovary syndrome [22, 47]. They aimed to determine the effect of metformin on breast size changes and breastfeeding success and found median duration of exclusive breastfeeding was slightly longer in the metformin group (4.5 vs. 3.9 months, *p* = 0.08) with no difference in total duration of any breastfeeding (8.8 vs. 8.5 months, *p* = 0.59) [47]. Subsequently, in 2015, Berens et al. performed a secondary analysis of a pilot randomized controlled trial using postpartum metformin versus placebo for weight loss in participants with gestational diabetes [46, 57]. In their secondary analysis, they aimed to determine the potential impact of postpartum metformin on breastfeeding outcomes during hospitalization and 3 and 6 weeks postpartum [46]. The overall rates of breastfeeding in this study were low with only 1 in 10 participants breastfeeding at 6 weeks postpartum and given their small cohort size of 43 and 36 participants in the placebo and metformin groups, respectively, this study was not powered to determine a measured significance of difference [46]. More recently, another pilot randomized trial was performed to determine the feasibility and acceptability of postpartum metformin to augment low milk supply among participants with low milk production and at least one measure of insulin resistance such as gestational diabetes, pregestational diabetes, or polycystic ovarian syndrome [45]. This study enrolled a total of 15 participants (2:1 randomization treatment to placebo) and found no difference with postpartum metformin and milk supply or breastfeeding outcomes [45].

Our results from a robust randomized controlled trial are consistent with the findings reported in the literature. Although metformin has been hypothesized to improve breastfeeding physiology by decreasing insulin resistance, ongoing evidence suggests no association between metformin and breastfeeding success albeit most of these studies are underpowered. From a clinical perspective, antepartum metformin should not be recommended solely for breastfeeding optimization in patients with type 2 diabetes or diabetes diagnosed in early pregnancy.

Although this secondary analysis did not demonstrate an effect of metformin on breastfeeding outcomes, our findings provide important information regarding breastfeeding outcomes and challenges among patients with diabetes. Among those who stopped breastfeeding or transitioned to formula feeding, only 18.5% of participants in the metformin group and 11.4% of participants in the placebo group responded that they had met their breastfeeding goals suggesting that the early cessation was not congruent with their intentions. Despite our negative findings, breastfeeding remains a challenging obstacle for patients with diabetes. The challenge with diabetes and breastfeeding is likely multifactorial since breastfeeding success has been correlated to multiple factors also present in patients with diabetes such as obesity, hypertensive disorders, cesarean delivery, NICU admission, and neonatal delivery complications. Additionally, socioeconomic factors such as paid maternity leave, need to return to work, and family and social support would play an important role in sustained breastfeeding at 30 days postpartum. While we cannot comment on the socioeconomic status of our participants directly, almost 74% in each arm were utilizing public insurance, a proxy for socioeconomic status, which is higher than expected.

Studies have already started looking toward tele‐visit and text message–based lactational support services as a means to improve breastfeeding rates and outcomes in the fourth trimester [58, 59, 60, 61]. However, our findings suggest that among those with difficulty sustaining breastfeeding, the perceived greatest contributors are maternal milk supply, neonatal latching, and babies not being satisfied by breastmilk alone. While these challenges may be addressed by educational interventions aimed at reassuring patients, it remains unclear whether tele visits or text messages alone would sufficiently address this. Future studies should focus on education to reassure patients as to the sufficiency of their supply, interventions to improve milk supply, and consideration for extended outpatient lactation consultative services.

As a randomized, double‐blinded, placebo‐controlled clinical trial with secondary analysis of data collected prospectively, there are multiple strengths to our study. To our knowledge, this is the largest placebo‐controlled trial used to assess the use of metformin in pregnancy and breastfeeding outcomes. We collected detailed breastfeeding information during pregnancy, immediately after delivery in the hospital, and at 30 days postpartum. Given the multicenter study design, our population is racially, ethnically, and geographically diverse with good generalizability. We had an overall high initial survey response rate of 80.9% and 78.3% in the metformin and placebo groups, respectively. Despite decreased responsiveness on the 30‐day postpartum survey, our follow‐up was still relatively robust. Additionally, our exposure groups did not have significant baseline differences and were similar with respect to confounders for breastfeeding (Table 1).

Our study does have limitations. First, the study was halted early for metformin futility for the primary study's composite outcome which limited the data collected for secondary analyses. We had limited power to show smaller differences in breastfeeding rates (e.g., 315 participants per group would be needed to statistically show a 10% difference in breastfeeding rates assuming a baseline rate of 67%). Additionally, the rate of breastfeeding in our population was much higher than expected from our a priori power calculations and thus, it is possible that benefits from metformin were unable to be demonstrated given the high baseline rate. Second, the COVID‐19 pandemic may have altered breastfeeding practices resultant from higher stress, isolation precautions, and lack of lactational support. Third, adherence to study medication was self‐reported instead of pill counting, which is beneficial for generalizability but may have limited our ability to assess the impact of metformin on breastfeeding due to treatment compliance. We do not know how each participant took metformin or placebo or for how long and we did not restrict whether primary providers utilized metformin in the postpartum period for management of diabetes. A shorter duration of metformin, poorer compliance to metformin versus placebo, or disproportionate metformin use postpartum would bias our findings toward the null. Specifically, patients may have been using metformin in the postpartum period in both the treatment and placebo groups, which would result in comparing antepartum plus postpartum metformin (treatment group) to just postpartum metformin (placebo group). Since we do not have robust studies for postpartum metformin and breastfeeding outcomes, it is possible that the use of postpartum metformin affected the breastfeeding outcomes. Fourth, since our study was performed across multiple clinical sites there may have been heterogeneity within each sites breastfeeding protocols, baby‐friendly status, and quality metrics to assess breastfeeding in the immediate newborn period such as PC‐05 measures that may have affected our findings. Finally, it is possible breastfeeding intentions changed between 24 and 30 weeks and by the time of delivery, however, this would be expected in both groups equally.

## 5 CONCLUSION

Our data suggests no association between metformin use during pregnancy and breastfeeding patterns among participants with type 2 diabetes or diabetes diagnosed in early pregnancy. Therefore, antepartum metformin should not be routinely prescribed to this population with the goal of improving breastfeeding success. As the breastfeeding rates in the obstetric population with diabetes are typically low, future studies should focus on educational initiatives and methods to improve sustained breastfeeding in this population.

## CONFLICT OF INTEREST STATEMENT

The authors declare no conflicts of interest.

## Supporting information



Supporting information

Supporting information
