# Supplementary material for: Breastfeeding patterns among parturients with diabetes: A secondary analysis of the MOMPOD randomized clinical trial
Source: Pregnancy (Hoboken). 2025 Jan 28;1(1):e12040. doi: 10.1002/pmf2.12040 (PMC12079847; doi:10.1002/pmf2.12040)
Supplement: Supplementary file 2 [file PMF2-1-e12040-s002.docx]

**Supplementary Figure 1. Prenatal Feeding Intention Survey**
